# Intraoperative ultrasonography assistance for minimally invasive repair of the acute Achilles tendon rupture

**DOI:** 10.1186/s13018-020-01776-6

**Published:** 2020-07-11

**Authors:** Yang Yongliang, Jia Honglei, Zhang Wupeng, Xu Shihong, Wang Fu, Wang Bomin, Li Qinghu, Wang Yonghui, Han Shumei

**Affiliations:** 1 Department of Trauma and Emergency, Shandong Provincial Hospital affiliated to Shandong University, No. 324 of Jingwu Road, 250021 Jinan, People’s Republic of China; 2 Department of Trauma and Emergency, Shandong Provincial Hospital affiliated to Shandong First Medical University, No. 324 of Jingwu Road, 250021 Jinan, People’s Republic of China; 3 Department of Oncology, Shandong Cancer Hospital and Institute, Shandong First Medical University and Shandong Academy of Medical Sciences, Jinan, 250021 China

**Keywords:** Intraoperative ultrasonography, Minimally invasive, Acute Achilles tendon rupture, Sural nerve injury

## Abstract

**Background:**

Minimally invasive repair is a better option for Achilles tendon rupture with low re-rupture and wound-related complications than conservative treatment or traditional open repair. The major problem is sural nerve injury. The purpose of this study was to evaluate the effect and advantage of the intraoperative ultrasonography assistance for minimally invasive repair of the acute Achilles tendon rupture.

**Methods:**

A retrospective study was performed on 36 cases of acute Achilles tendon rupture treated with minimally invasive repair assisted with intraoperative ultrasonography from January 2015 to December 2017. The relationship of the sural nerve and small saphenous vein was confirmed on the preoperative MRI. The course of the small saphenous vein and the sural nerve was identified and marked by intraoperative ultrasonography. The ruptured Achilles tendon was repaired with minimally invasive Bunnell suture on the medial side of the small saphenous vein (SSV).

**Results:**

All patients were followed up for at least 12 months. No sural nerve injury or other complications was found intraoperatively and postoperatively. All the patients returned to work and light sporting activities at a mean of 12.78 ± 1.40 weeks and 17.28 ± 2.34 weeks, respectively. The Mean American Orthopaedic Foot & Ankle Society (AOFAS) scores improved from 59.17 ± 5.31 preoperatively to 98.92 ± 1.63 at the time of 12 months follow-up. There was a statistically significant difference (*P* < 0.001). No patient complained of a negative effect on their life.

**Conclusions:**

The minimally invasive repair assisted with intraoperative ultrasonography can yield good clinical outcomes, less surgical time, and less complications, especially sural nerve injury. It is an efficient, reliable, and safe method for acute Achilles tendon (AT) rupture.

## Background

Achilles tendon (AT) rupture is a common injury of the foot and ankle, which mostly occurs in the 30–40-year age group, and the incidence is increasing in recent years [1]. The optimal treatment for acute Achilles tendon rupture is still controversial since now. Treatment can be classified into conservative and surgical types. Conservative management using a short-leg resting cast or brace in an equinus position was shown to be associated with an increased re-rupture incidence of, about 10–12% compared with about 4–5% in patients undergoing surgery [2, 3]. Surgical treatment can be subdivided into open sutures with or without augmentation, minimally invasive, and percutaneous repairs. In comparison with conservative management, surgical repair can decrease the tendon re-rupture rate, and get earlier functional treatment, less calf atrophy, and stronger push off [4]. However, the possible surgical complications included wound infection and sural nerve (SN) injury.

Percutaneous repair, first described by Ma and Griffith [5] in 1977, has the advantage of less chance of wound breakdown, but this technique has two potential risks. The first risk is sural nerve injury owing to the close anatomic neighborhood with the path the needle takes and the nerve. The recent literatures reported a range of 0–27% of sural neuritis after percutaneous repair [2, 3, 6–9]. The second risk is an inability to assess the quality of the tenodesis because this technique does not use a surgical incision that allows exposure of the tendon ends. Kakluchi [10] described a new method that combined the advantages of open and percutaneous techniques in 1994. Assal et al. [11] developed a device called the Achillon to improve on the method reported by Kakluchi. They conducted a preliminary study using 16 fresh cadavers and reported their satisfactory clinical results of a multicenter, prospective study of 87 patients in 2002 [11]. Based on the Achillon method, Chen et al. [12] conducted a new method, which is called channel-assisted minimally invasive repair (CAMIR), to avoid sural nerve injury and yield essentially identical clinical and functional outcomes compared with open repair. But these devices (Achillon or CAMIR) are too expensive to apply beyond some patients’ affordability in our hospital. Some authors reported that the ultrasound examination was used to determine the passage of the sural nerve relative to the Achilles tendon or the small saphenous vein (SSV) [13, 14]. Therefore, intraoperative ultrasonography was used to assist minimally invasive repair of acute AT rupture [15–17]. In this present study, we assessed the outcomes of a minimally invasive repair assisted with intraoperative ultrasonography in patients diagnosed as acute AT rupture, and evaluated the effect and advantage of the intraoperative ultrasonography assistance for minimally invasive repair of the acute Achilles tendon rupture. We used ultrasonography to identify the small saphenous vein and sural nerve and marked them in advance to achieve the purpose of minimally invasive treatment without damage to the sural nerve. This study mainly carries on research in this aspect.

## Materials and methods

**Fig. 1:**
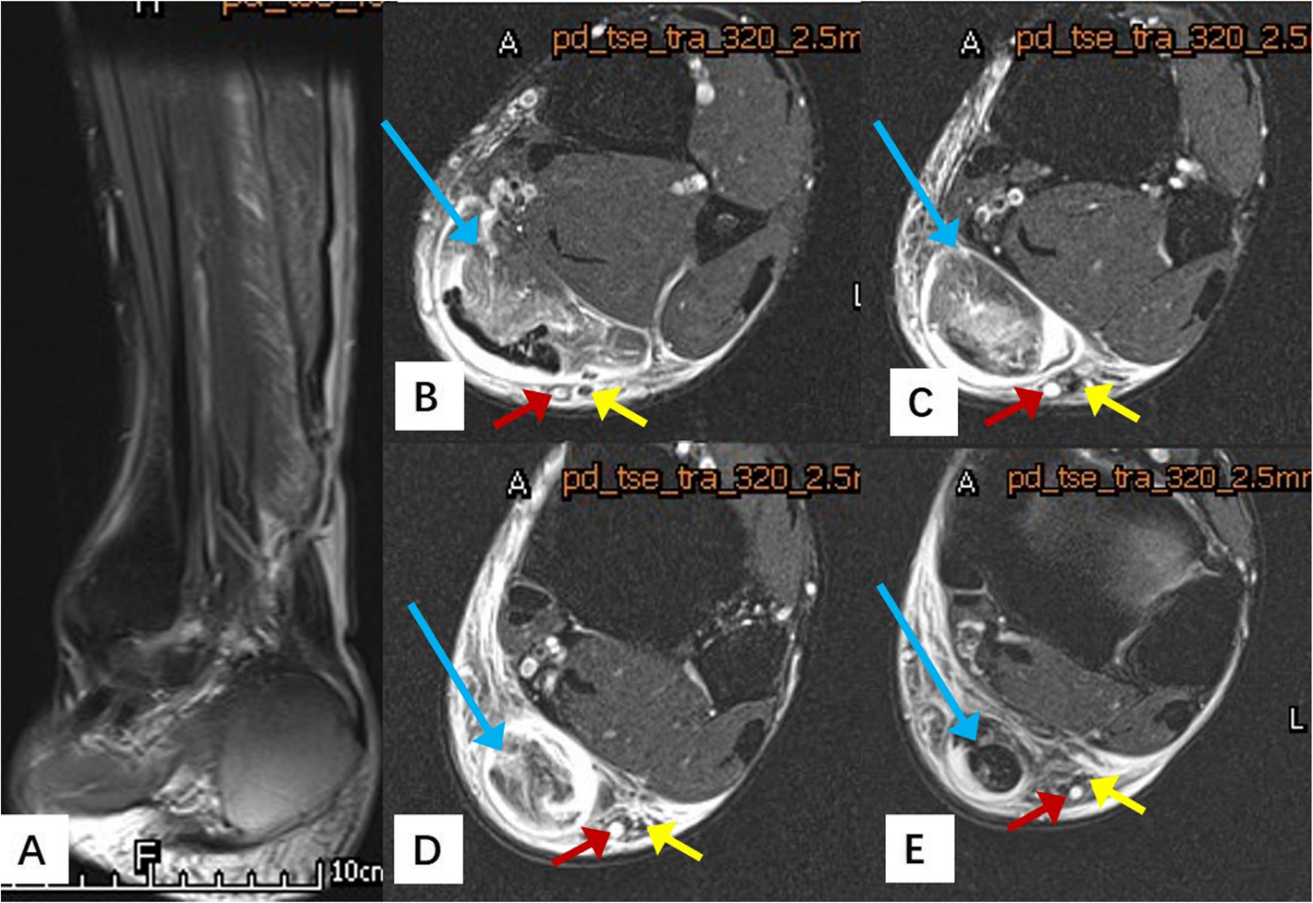
A male patient, 35 years old, with the Achilles tendon rupture in the basketball game. **a**. Magnetic resonance imaging scan shows an Achilles tendon rupture with retraction and gapping between the tendon ends on the sagittal image. **b–e**. Magnetic resonance imaging scan shows the relationship of the tendon, the sural nerve (SN), and the small saphenous vein (SSV) on the cross-sectional images. The blue arrow shows the ruptured tendon; the red arrow shows the small saphenous vein; the yellow arrow shows the sural nerve

From January 2015 to December 2017, 36 consecutive patients diagnosed as acute AT rupture were treated with minimally invasive repair assisted with intraoperative ultrasonography. The AT ruptures were left in 15 cases and right in 21 cases. The inclusion criteria consisted of an acute close AT rupture with no calcaneal fracture, a palpable gap between the ruptured ends, a positive Thompson test, and a distal stump more than 2 cm from the insertion confirmed by ultrasonography. Patients with re-ruptures, Achilles rupture of more than 3 weeks, previous history of local surgery or injury, history of steroid injection, chronic diseases, a distal tendon stump less than 2 cm or more than 8 cm from its insertion [15], were excluded from this study. The patients’ characteristics were listed in Table 1. The career of the patients almost included the athlete, the amateur, or the sportsman, who were more easily suffering from the AT rupture. The reason for injury was a sports injury in 36 patients, including basketball, football, badminton, and running. Simultaneously, MRI or ultrasonography was performed to confirm the AT rupture and the relationship of SSV and SN (Fig. 1).

**Table 1:** Characteristics of the patients

Variable	Study subjects (*n* = 36)
Age, years (mean ± SD)	35.4 ± 7.79
Male (*n*, %)	28 (77.78%)
Injured side (*n*, %)	
Left	15 (41.67%)
Right	21 (58.33%)
Cause of injury (*n*, %)	
Basketball	11 (30.56%)
Football	9 (25%)
Badminton	12 (33.33%)
Running	4 (11.11%)
Tear site (distance from the calcaneal insertion) (cm, mean ± SD)	4.72 ± 1.23

*SD* standard deviation

### Surgical methods

**Fig. 2:**
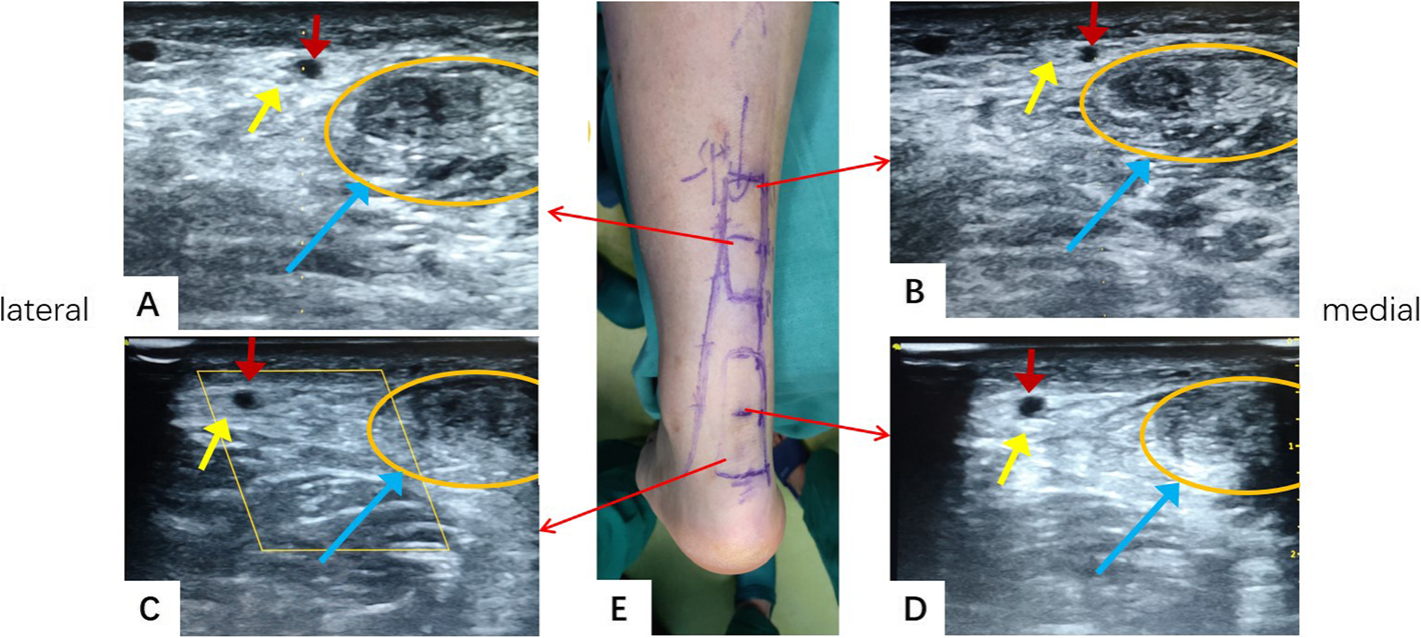
The intraoperative ultrasonography shows the relationship of the tendon, the SN, and the SSV at different levels from the insertion of the tendon on the calcaneus. It demonstrates that the SSV passes along the medial side of the SN. The distance between tendon and the SSV is shorter from the insertion of the tendon to the proximal side. The blue arrow shows the ruptured tendon; the red arrow shows the small saphenous vein; the yellow arrow shows the sural nerve

After the success of anesthesia, all patients were operated in a prone position without a tourniquet. None of the surgeons received any specific training from radiologists. The intraoperative ultrasonography was used to determine the location of the stump and to identify the lateral and medial edges of the tendon. The SN was identified from the lateral retro-malleolar groove near the SSV to the intersection with the lateral edge of the tendon. The SSV always passed along the medial side of the SN based on Eid, E. M.’s anatomical study [16]. It was easier to identify the location of the SSV than that of the SN for the surgeon with the intraoperative ultrasonography. The course of the SSV and the tendon was marked on the posterior skin of calf (Fig. 2).

A 2-cm vertical or horizontal skin incision was made at the level of a palpable gap in the substance of the ruptured Achilles tendon. The paratenon was identified and incised if it was still intact. The ruptured ends of the tendon were identified through palpation and visualization. After the avulsion of the AT stump was exposed clearly, the proximal and distal AT ends were grasped with artery forceps and brought out from the incision.

The proximal three longitudinal stab incisions were made on the lateral side of the tendon 5 cm, 3 cm, and 1 cm proximal to the palpable defect, and the other three incisions were made on the medial side of the tendon at the corresponding position. The proximally lateral stab incision was close to the SN and the SSV. In order to avoid damage to the SN, this lateral incision was medial to the marked lines of the SSV, which was defined by intraoperative ultrasonography before surgery. Four longitudinal stab incisions on the distal side of the tendon were just proximal to the insertion of the tendon on the calcaneus.

**Fig. 3:**
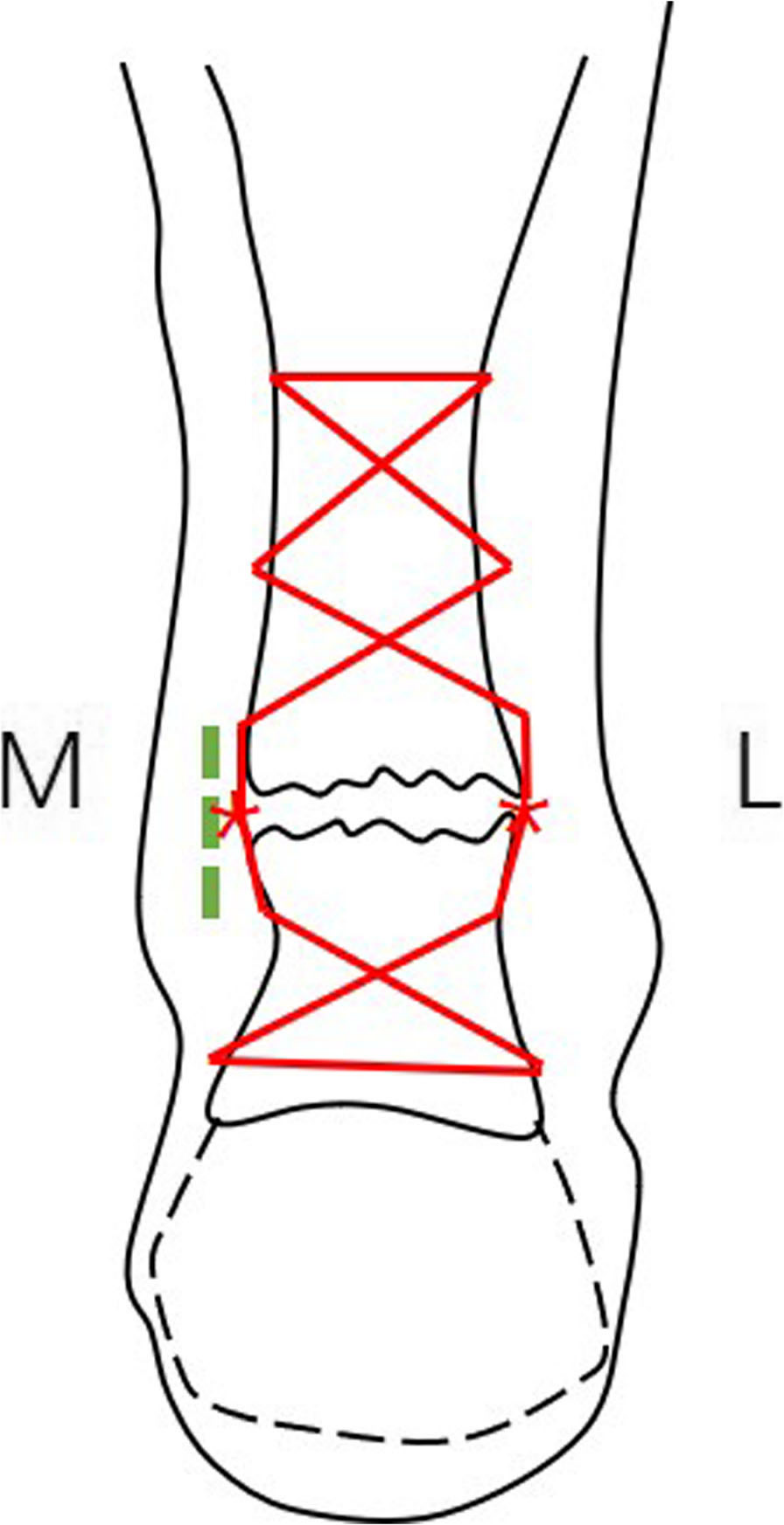
Minimally invasive modified Bunnell suture with knots outside the tendon. Green dotted lines indicate placement of the incision. Red lines indicate the sutures holding both parts of the rupture. M: medial; L: lateral

**Fig. 4:**
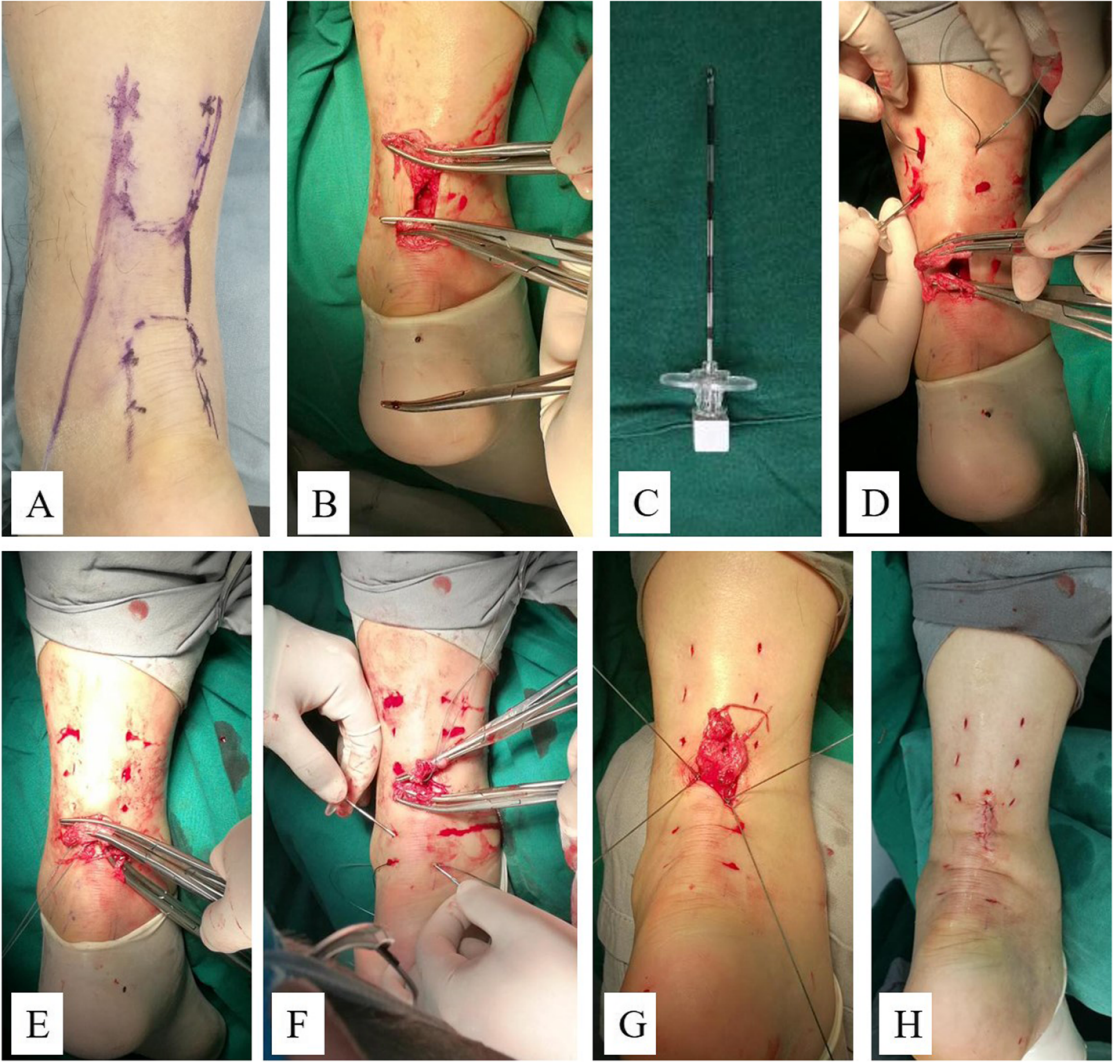
The procedure of minimally invasive repair. **a** The location of the torn tendon and the course of the SSV were identified and marked after anesthesia. **b** A small incision was made at the level of the palpable gap and the proximal and distal Achilles tendon ends were grasped with artery forceps and brought out from the incision. **c** The trocar of epidural anesthesia needle was used as the guide needle. **d** Symmetrical mini-incisions, medial and laterally were performed along the course of the proximal stump tendon at a distance of approximately 2 cm. The first No.2 ETHIBOND suture was inserted from the proximally lateral incision (this incision was made medially to the SSV) to the opposite one, each of the ends was passed to the diagonal opposing side guided by the trocar of epidural anesthesia needle. **e** The two ends of the first suture were pulled out of the incision on the medial side of the torn tendon. **f** The second No.2 ETHIBOND suture was inserted into the distal stump tendon with the same procedure. **g** The ends of the two sutures were knotted on bilateral sides of the torn tendon. **h** The ruptured tendon was repaired with minimally invasive suture

The percutaneous repair method was modified on the basis of the Bunnell suture (Fig. 3). A No.2 ETHIBOND suture (polyester unabsorbable suture, Johnson & Johnson, USA) was guided through the trocar of the epidural anesthesia needle, which was passed transversely between the proximal stab incisions through the bulk of the tendon. Each of the ends was then passed distally from just proximal to the transverse passage through the bulk of the tendon to pass out of the diagonally opposing stab incision with the trocar of the epidural anesthesia needle. Each of the ends was passed to the diagonal opposing side with the same procedure and pulled out of the incision on the medial side of the torn tendon. In turn, the distal side of the tendon was stitched with the other No.2 ETHIBOND suture by the same method, and the ends of the distal suture were pulled out of the same incision over the torn tendon. The two ends of the proximal ETHIBOND suture tied with the two ends of the distal suture tightly on both sides of the torn tendon at the flexion position of ankle and knee joints. The two ends of the torn tendon were sutured with 3-0 absorbable sutures (Johnson & Johnson, USA). The paratenon was repaired with 2-0 absorbable sutures (Johnson & Johnson, USA), then the incision was closed with intradermal suture (Fig. 4).

### Postoperative care and rehabilitation

After the wound closure, a short leg dorsal cast was applied to the patient in equinus position at 20^o^ to 30^o^. For the first 3 weeks, an active range of motion exercise (for the toe, knee, and hip joint) and muscle-strengthening exercises were recommended with the aim of preserving muscle strength. The crutches were used to ambulate for the patients. The casts were removed at the end of the sixth postoperative week. Then a walker boot was given to maintain the ankle at the same flexion degree for another 4 to 6 weeks. The patients were told to walk with the crutches and remove the boot until neutral flexion was achieved. After removal of the boot, active plantar flexion exercises were started and normal shoes were permitted to wear. The active resistive and stretching exercises were started. Sports similar to jogging were allowed at 12 weeks postoperatively.

### Follow-up and data collection

The mean follow-up time was 16.69 ± 4.04 months (range, 12–24 months). Follow-up examinations of the patients were performed according to a standard protocol by the same surgeon that had performed the surgical procedure at 3, 6, and 12 weeks postoperatively. The evaluations were performed in a standardized fashion by two examiners, who were not the operating surgeons and all patients were evaluated during the 1-year follow-up period. American Orthopaedic Foot & Ankle Society (AOFAS) scale scores were used to evaluate patients preoperatively and 12 months follow-up. The significance threshold was defined as 0.05.

### Perioperative index

Thirty-six patients (28 males and 8 females) were included in this study. The perioperative index was listed in Table 2**.** The mean age of these patients was 35.4 ± 7.79 years (range, 18–52 years). The average period from injury to operation was 1.83 ± 0.60 days (range, 1–5 days). The mean tear site of the distance from the calcaneal insertion was 4.72 ± 1.23 cm (range, 3–8 cm). The mean surgical time was 22.11 ± 5.29 min (range, 15–40 min). The mean blood loss of surgery was 18.47 ± 6.74 ml (10–40 ml) without the use of the tourniquet. The mean length of incision was 2.12 ± 0.32 cm (range, 1.6–3.0 cm). The mean days of hospitalization were 3.17 ± 0.81 days (range, 2–6 days).

**Table 2:** Perioperative index

Variable	Study subjects (*n* = 36)
Time from injury to surgery (days)	1.83 ± 0.60
Operation time (min)	22.11 ± 5.29
Mean time of hospitalization (days)	3.17 ± 0.81
Length of incision (cm)	2.12 ± 0.32
Blood loss of surgery (ml)	18.47 ± 6.74
Follow-up (months)	16.69 ± 4.04
Complications	0
3-month AOFAS	92.42 ± 1.38
12-month AOFAS	98.92 ± 1.63
Time to work (weeks)	12.78 ± 1.40
Time to light sports (weeks)	17.28 ± 2.34

*AOFAS* American Orthopaedic Foot & Ankle Society

## Results

In this study, the preoperative AOFAS ankle-hindfoot scores improved from 59.17 ± 5.31 (range, 52–72) to 98.92 ± 1.63 (range, 95–100) at the time of 12 months follow-up. This was a statistically significant difference (*P* < 0.001). All patients could return to their work and their light sporting activities at the time of 12.78 ± 1.40 weeks (range, 10–16) and 17.28 ± 2.34 weeks (range, 14–24) postoperatively, respectively. No difference was found between the injured extremity and healthy extremity in the “single extremity jump-landing” test. No patient complained that this minimally invasive operation had a negative effect on their social or professional life.

None of the patients developed superficial or deep infections in the surgical site and there was no wound healing problem in all patients. No sensory loss in the foot or ankle associated with SN injury or no re-rupture was observed among patients in this group during the surveillance period. And no clinical evidence of deep vein thrombosis or pulmonary embolism was observed in this study.

## Discussion

Most recent meta-analyses have reported that a significantly lower risk of re-rupture with open repair techniques compared with conservative treatment, but the risks of wound-related complications and skin adhesions persist [1, 3, 17, 18]. Percutaneous and minimally invasive techniques have several advantages to reduce these complications. For example, the risk of wound problems is lower than the open repair, and the risk of re-rupture is lower compared with the conservative treatment [17, 19]. It was known that there was a major problem of sural nerve injury during minimally invasive suturing of the AT rupture. Ma and Griffith firstly described the percutaneous suture technique based on Bunnell sutures for AT rupture in 1977 [5]. However, Klein reported a 13% rate of iatrogenic sural nerve injury with the Ma–Griffith technique for percutaneous repair of fresh ruptured tendon Achilles [20]. Haji reported the risk of sural nerve injury was 10.5% in 38 patients who underwent repair of acute AT rupture using a modified Ma and Griffith percutaneous technique [21]. Although there have been no clinical reports that the Achillon technique produces sural nerve injury, Porter’s cadaveric study showed a 27% risk of sural nerve violation during percutaneous Achilles tendon repair using Achillon device [6] because the sural nerve displayed a highly variable anatomical course. The other minimally invasive technique, which was called Channel-assisted minimally invasive repair (CAMIR) and designed by Dr. Chen from China [12], could minimize the possibility of sural nerve injury and yield essentially identical clinical and functional outcomes compared with open repair. But this CAMIR device is so expensive that limits its clinical application. The intraoperative ultrasound was used to decrease the risk of sural nerve injury to assist the minimally invasive repair of the acute AT rupture.

The sural nerve (SN) is a sensory nerve in the lower extremity which branches to supply the skin on the distal posterolateral third of the lower limb. Typically, the SN is sought along the course of the small saphenous vein (SSV), where it is complete after the union of the medial sural cutaneous nerve (MSCN) and the peroneal communicating nerve (PCN) [22]. Kammar et al. reported that the mean distance between the sural nerve and the Achilles tendon was 21.48, 11.47, 5.8, and 0.81 mm lateral to the tendon as measured at the insertion and 4, 8, and 11 cm proximally, respectively [13]. Falvin et al. have proposed a clinical method to locate the sural nerve before the surgical procedure. In their study, the average of the error of distance of the method was 2.5 mm, calculated as the distance between the clinical measurement and the ultrasound images [23]. In Zappia’s cadaveric study [24], the average distance between the suture and the sural nerve was 2.1 mm, less than the result (2.5 mm) reported by Flavin et al. [23].

Intraoperative high-resolution real-time ultrasound can be of assistance during percutaneous repair of AT rupture, with no complication related to the ultrasonography. In Giannetti et al. study, no surgery-related complications, such as wounds or deep infections, sural nerve injury, were detected at follow-up^7^. The ultrasonography control was performed to notice the position of the needle during the needle puncture. But it must be considered that the use of high-resolution real-time ultrasonography does require the presence of an experienced imaging specialist and its more time and cost consuming [25]. In our study, there was no SN injury, re-rupture, superficial or deep infections, or wound healing problem among patients, which was the result from the ultrasonography assistance and the minimal invasion.

The relationship of the sural nerve and the small saphenous vein may be helpful for the Orthopaedic surgeon to repair the AT rupture with a minimally invasive technique. Eid et al dissected anatomical variations of the sural nerve and its role in clinical and surgical procedures in 24 Egyptian legs and feet, they found that the small saphenous vein passed along the medial side of the sural nerve in all cases [16]. Therefore, the suture needle was punctured through the medial side of the SSV with the Bunnell suture method as a result the risk of sural nerve injury was avoided theoretically. It is difficult to accurately locate the SSV and the SN intraoperatively for an Orthopaedic surgeon. According to Eid, E. M. results, the course of the SSV instead of the SN, can be detected with the intraoperative ultrasonography because the SSV is easier to be found than the SN with the intraoperative ultrasonography for an Orthopaedic surgeon without an experienced imaging technique. Therefore, the suture needle was punctured through the medial side of the SSV to avoid the possibility of the sural nerve injury. In our study, the rupture of the Achilles tendon and the course of the SSV were detected with the intraoperative ultrasonography after anesthesia on prone position. The SN injury was not found in our cases after surgery with the Bunnell percutaneous repair method assisted with the intraoperative ultrasonography.

Some studies reported that open surgery around the Achilles tendon had a wound-related complication rate of between 8.2% and 34.1% [26–28], of which at least half are due to infection [29]. The Achilles tendon is more susceptible to infection than other parts of the ankle because of its poor blood supply [30]. Paavola et al. reported that the use of tourniquets might be detrimental to wound healing and the retraction of soft tissue during surgery might increase the risk of wound infection [31]. The percutaneous repair of the Achilles tendon is known to reduce the risk of wound site infection compared with open surgery methods [32], but sural nerve injuries may be a risk factor with this treatment method. The tourniquet was not used in the case with this minimally invasive method. The paratenon, which is located between the tendon and the skin, provides a valuable blood supply to the repaired tendon and avoids skin tethering to the AT, it also prevents superficial infection spreading into the deep layers. A 2.0-cm vertical incision was made in the paratenon to display the ruptured Achilles tendon, the paratenon was completely closure after the tendon was repaired. The suture knots were located outside the repaired tendon. All of these conditions protect the blood supply to the AT and promote tissue healing. In our study, there were no wound-related complications and no case of re-rupture with our minimally invasive repair method.

Although we achieved satisfactory and good clinical outcomes, there were several limitations in this study. First, this is a retrospective non-controlled study with a relatively small number of patients result in selective bias. The number of patients was not large enough to provide a valid conclusion. Second, the suture method in this study is based on the Bunnell suture and modified, the suture knots located outside of the repaired tendon. Whether this technique can achieve good strength of AT repair needs to be further biomechanically compared with the open repair and other minimally invasive repair methods. Finally, it should be proven whether this technique influences the blood supply of the Achilles tendon.

## Conclusion

The minimally invasive repair assisted with intraoperative ultrasonography can yield good clinical outcomes, less surgical time, and less complications, especially sural nerve injury. The technique can save the cost of surgery with the comparison of the Achillon device. In our study, the surgeon does not accept the radiological training, it is easy to identify the course of the SSV by intraoperative ultrasonography. The suture needle punctures the medial side of the SSV to avoid SN injury. This technique effectively reduces the risk of sural nerve injury in this study. This study shows that minimally invasive repair assisted with intraoperative ultrasonography is an efficient, reliable, and safe method for acute AT rupture.

## Data Availability

The datasets used and analyzed during the current study are available from the corresponding author on reasonable request.
